# Alopecia Areata in a Patient Undergoing Treatment for Secondary Syphilis: A Diagnostic Challenge

**DOI:** 10.7759/cureus.103005

**Published:** 2026-02-05

**Authors:** Diego I Mendez-Villanueva, Ana Martinez, Diana Castro, Nicolas Opazo, Diego Guarda

**Affiliations:** 1 Dermatology, Universidad de Santiago de Chile, Santiago, CHL; 2 Internal Medicine, Universidad Andrés Bello, Santiago, CHL; 3 Dermatology, Hospital El Pino, Santiago, CHL; 4 General Medicine, Clínica Andes Salud, Puerto Montt, CHL

**Keywords:** alopecia areata, anchoring bias, syphilis, syphilitic alopecia, trichoscopy

## Abstract

A 17-year-old male with a recent history of incarceration and a diagnosis of secondary syphilis presented for follow-up three months after receiving appropriate antibiotic treatment. His venereal disease research laboratory (VDRL) titers showed a favorable decline (from 1:16 to 1:4); however, he reported new-onset patchy scalp alopecia. Clinically, the lesions raised suspicion for syphilitic alopecia (SA). Nevertheless, trichoscopy revealed hallmark findings of alopecia areata (AA), including exclamation mark hairs and black dots, while lacking specific signs of SA, such as an erythematous background. HIV screening was negative. The patient showed significant hair regrowth after six weeks of topical clobetasol. This case highlights that AA can clinically mimic SA, even in patients with active or recent syphilis, and underscores the value of trichoscopy in preventing misdiagnosis and avoiding anchoring bias.

## Introduction

Non-scarring alopecia has a broad differential diagnosis. Among its causes, alopecia areata (AA) and syphilitic alopecia (SA) may present with similar clinical features, including multiple well-defined patches of hair loss [1,2]. SA is a recognized manifestation of secondary syphilis, while AA is a common autoimmune disorder affecting hair follicles. In clinical practice, AA can act as a mimicker of SA, especially in patients with recent or concurrent syphilis [2,3].

Given the rising prevalence of syphilis and the overlap in clinical presentation, distinguishing between AA and SA is essential to avoid misdiagnosis. Trichoscopy has emerged as a valuable tool in identifying key dermoscopic features that aid in differentiation [1,4,5].

Misdiagnosing AA as SA may lead to unnecessary repeated antibiotic courses and psychological distress, while failing to recognize AA delays appropriate corticosteroid therapy. AA is characterized by the collapse of the hair follicle's immune privilege, a process that can be triggered by systemic stressors.

## Case presentation

A 17-year-old male with a recent history of incarceration was evaluated in dermatology follow-up for secondary syphilis. Three months earlier, he received two doses of benzathine penicillin G (2.4 million units IM, one week apart). This regimen was chosen following national guidelines for early syphilis.

**Figure 1:**
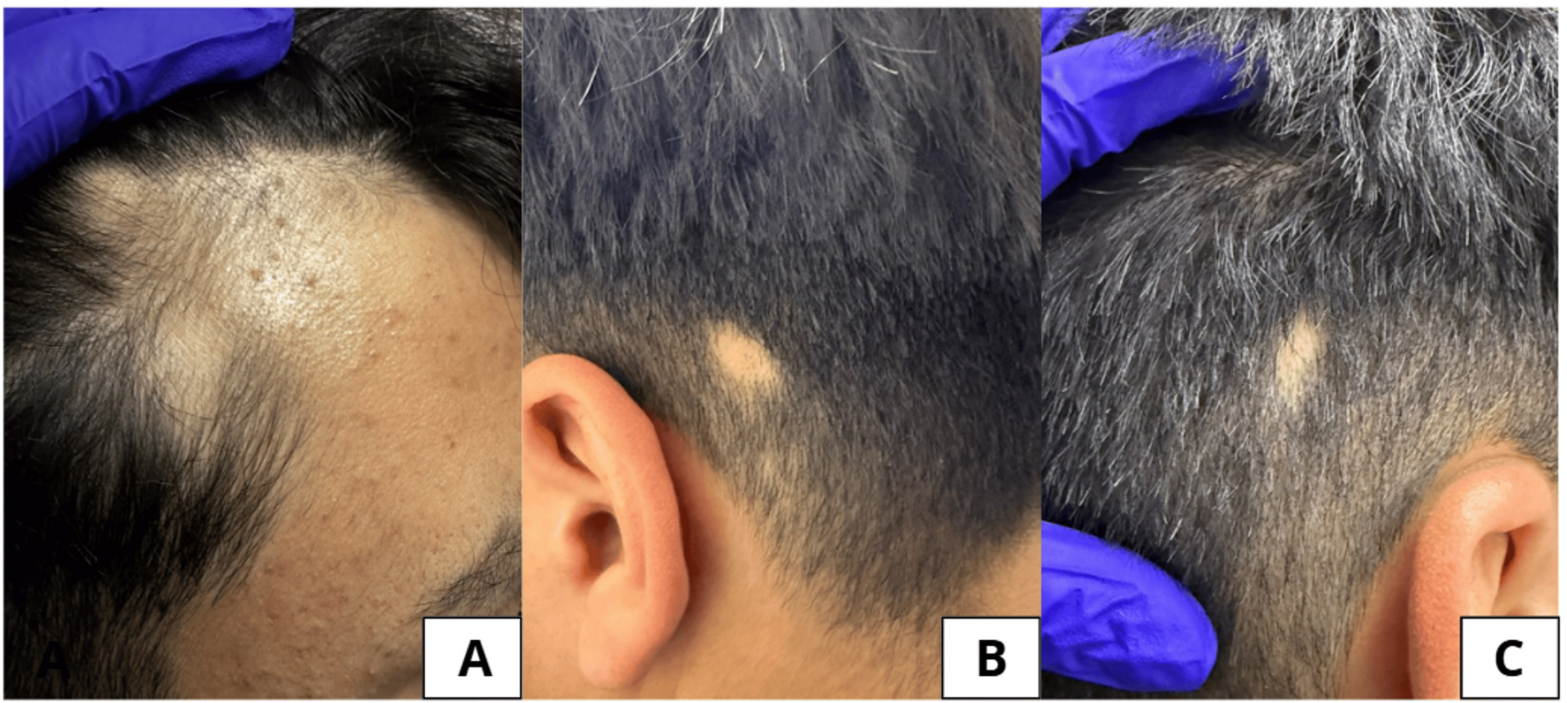
Clinical images of the patient showing alopecic patches consistent with alopecia areata. (A) Frontal scalp with a well-demarcated patch showing smooth, hairless skin without scaling or erythema; and (B, C) occipital and retroauricular areas with sharply defined, circular alopecic plaques.

At follow-up, the venereal disease research laboratory (VDRL) titer had decreased to 1:4 (from an initial 1:16), representing a two-dilatation (four-fold) decline. In the context of syphilis management, a four-fold decrease in non-treponemal titers within six to 12 months is defined as an adequate biochemical response to treatment (expected response: ≥4-fold titer decrease). Screenings for HIV (Ag/Ab) and other sexually transmitted infections (STIs) remained non-reactive (reference range: non-reactive). Despite this favorable serological progress, the patient reported a two-week history of asymptomatic patchy hair loss on the scalp. Physical examination revealed multiple sharply demarcated, non-inflammatory, non-scarring alopecic patches (Figures 1A-1C). Given his history, SA was considered.

**Figure 2:**
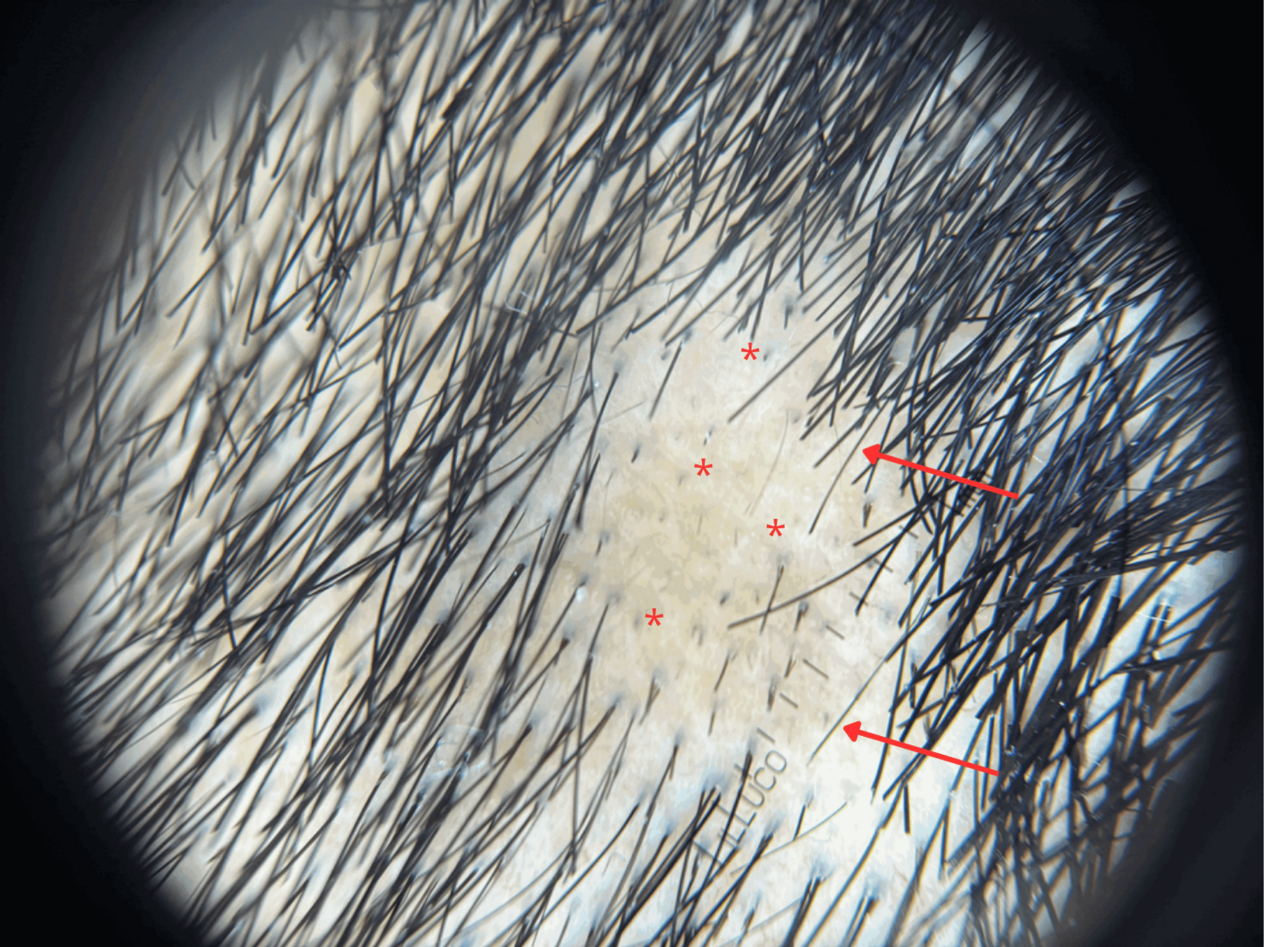
Trichoscopic image of the scalp showing characteristic findings of alopecia areata. Characteristic findings of alopecia areata are highlighted - red arrows indicate "exclamation mark hairs" (tapering toward the proximal end), and red asterisks (*) mark "black dots" (cadaverized hairs) resulting from hair shaft fracture at the scalp level. The absence of an erythematous background or perifollicular scaling helps differentiate this presentation from syphilitic alopecia.

Trichoscopy, however, showed exclamation mark hairs, black dots, short vellus regrowing hairs, and broken hairs; findings consistent with AA and not typical of SA (Figure 2) [1,4]. The absence of "coiled hairs" or follicular hemorrhages helped exclude trichotillomania. Furthermore, the four-fold decline at the three-month mark represents an early and favorable serological response, further supporting the exclusion of active syphilitic alopecia at the time of hair loss.

The patient was treated with topical clobetasol propionate 0.05% cream applied twice daily. At the six-week follow-up, he showed significant clinical improvement with over 75% hair regrowth in the previously affected patches. The patient provided written informed consent for the publication of this case report and the associated clinical and trichoscopic images.

## Discussion

This case illustrates how alopecia areata (AA) can clinically mimic syphilitic alopecia (SA), particularly in patients with a recent history of secondary syphilis. Both conditions represent forms of non-scarring alopecia that may present with overlapping clinical features. In classical dermatology, SA is often regarded as a manifestation of late secondary syphilis, typically appearing months after the initial infection or during recurrent episodes [6]. While SA characteristically presents with a "moth-eaten" pattern, it can also manifest as patchy or diffuse loss, further complicating the differential diagnosis [5,7]. In contrast, AA is an autoimmune disorder characterized by the collapse of the hair follicle’s immune privilege, leading to localized cytotoxic infiltration [8].

The main clinical and trichoscopic differences between these two entities are summarized in Table 1. Trichoscopy has become an essential diagnostic tool in this context. AA displays hallmark features, such as exclamation mark hairs, black dots, and broken hairs [1,8]. Recent studies highlight that exclamation mark hairs have a high discriminative value (area under the curve {AUC}: 0.816) for AA when compared to SA, which more frequently lacks these signs and may instead show non-specific findings like empty follicular openings, perifollicular scaling, or an erythematous background [1,2]. Indeed, Doche et al. noted that the specific combination of broken hairs, exclamation mark hairs, and black dots was observed exclusively in patients with AA [2].

**Table 1:** Comparative analysis of clinical and trichoscopic findings. Comparison parameters and trichoscopic significance are based on the diagnostic criteria for AA and SA described in references [1,2]. The statistical discriminative value for exclamation mark hairs (AUC: 0.816) is specifically attributed to the multivariate analysis by Tejapira et al. [1]. Pathophysiological background for both entities is supported by references [6,8]. AUC: area under the curve; VDRL: venereal disease research laboratory

Features	Alopecia areata (AA)	Syphilitic alopecia (SA)	Significance
Clinical pattern	Sharply demarcated, smooth circular patches	"Moth-eaten" (pathognomonic), patchy, or diffuse	Reflects localized autoimmune vs. systemic infectious patterns
Exclamation mark hairs	Highly prevalent	Extremely rare or absent	Highest discriminative value for AA (AUC: 0.816)
Black dots	Common in active phase	Rare	Indicates rapid destruction of the hair shaft
Yellow dots	Common (pure sebum)	Reddish-yellow dots	Reddish hue in SA indicates hemosiderin and inflammation
Background color	Normal or ivory/yellow	Erythematous or reddish-brown	Indicates active systemic inflammation in SA (p=0.008)
Vascular patterns	Non-specific	Dilated, tortuous, or arborizing vessels	Corresponds to the vasculitis-like process of syphilis
Pull test	Positive at the active margins	Often positive globally	Distinguishes localized activity (AA) from systemic effluvium (SA)
Serology	Unrelated to VDRL titers	Directly correlated with titers	High titers confirm SA; declining titers support AA

Clinical context remains paramount. AA typically has a sudden onset and is frequently triggered by psychological stress. In our patient, recent incarceration was identified as the primary psychosocial stressor. During the clinical interview, other potential triggers, such as family bereavement, thyroid dysfunction, or recent acute febrile illnesses, were screened and excluded. This supports the hypothesis that the neuroimmune response was likely triggered by the stress of his legal situation [8]. Furthermore, therapeutic response serves as an ex juvantibus diagnostic clue - AA responds to corticosteroids, whereas SA requires antibiotic therapy. The patient's significant regrowth following topical clobetasol, combined with declining VDRL titers, confirmed the diagnosis of AA. This case underscores the importance of maintaining a high index of suspicion for primary dermatological disorders to avoid "anchoring bias" in patients with a known history of systemic infections like syphilis.

Although other forms of non-scarring hair loss, such as telogen effluvium, were considered, the localized nature of the patches and the specific trichoscopic markers pointed directly to AA. Our diagnostic conclusion is based on robust clinicodermoscopic correlation rather than histopathological confirmation, which was avoided to minimize invasive procedures.

## Conclusions

In patients with a history of syphilis, patchy alopecia should not be automatically attributed to the infection. This case illustrates that AA can mimic SA and underscores the role of trichoscopy in preventing anchoring bias. While these findings are illustrative of a single-case experience, they emphasize the importance of clinical-serological correlation in ensuring accurate diagnosis and avoiding unnecessary treatments.
